# Ectodomain Shedding by ADAM17 Increases the Release of Soluble CD40 from Human Endothelial Cells under Pro-Inflammatory Conditions

**DOI:** 10.3390/cells12151926

**Published:** 2023-07-25

**Authors:** Anton Klersy, Sören Meyer, Florian Leuschner, Thorsten Kessler, Markus Hecker, Andreas H. Wagner

**Affiliations:** 1 Department of Cardiovascular Physiology, Heidelberg University, 69120 Heidelberg, Germany; 2 Department of Cardiology, Angiology and Pneumology, Heidelberg University, 69120 Heidelberg, Germany; 3 Department of Cardiology, German Heart Centre Munich, Technical University of Munich, 80636 Munich, Germany; 4 DZHK (German Centre for Cardiovascular Research), Partner Site Munich Heart Alliance, 80636 Munich, Germany

**Keywords:** single nucleotide polymorphism, soluble CD40, biomarker, ADAM17, endothelial cells

## Abstract

Background: Homozygosity for the C allele of the −1T>C single nucleotide polymorphism (SNP) of the *CD40* gene (rs1883832) is associated with susceptibility to coronary heart disease (CHD), enhanced CD40 expression, and shedding. The disintegrin metalloprotease ADAM17 can cleave various cell surface proteins. This study investigates an association between ADAM17-mediated CD40 shedding and inflammation in CC genotype human endothelial cells. Methods: Human umbilical vein endothelial cells (HUVEC) carrying the CC genotype were stimulated with soluble CD40 ligand (sCD40L) or tumor necrosis factor-α (TNFα). Messenger RNA and protein expression were determined with standard methods. Levels of high sensitive c-reactive protein (hs-CRP), interleukin-6 (IL-6), and sCD40 in plasma samples from patients with CHD were assessed using ELISA. Results: ADAM17 surface abundance was elevated following stimulation with CD40L and TNFα just as its regulator iRhom2. Inhibition of ADAM17 prevented TNFα-induced sCD40 and soluble vascular cell adhesion molecule-1 release into the conditioned medium and reinforced CD40 surface abundance. Secondary to inhibition of ADAM17, stimulation with CD40L or TNFα upregulated monocyte chemoattractant protein-1 mRNA and protein. Levels of sCD40 and the inflammatory biomarkers hs-CRP and IL-6 were positively correlated in the plasma of patients with CHD. Conclusions: We provide a mechanism by which membrane-bound CD40 is shed from the endothelial cell surface by ADAM17, boosting sCD40 formation and limiting downstream CD40 signaling. Soluble CD40 may represent a robust biomarker for CHD, especially in conjunction with homozygosity for the C allele of the −1T>C SNP of the *CD40* gene.

## 1. Introduction

In various systemic inflammatory diseases, such as diabetes or atherosclerosis, an eminent role for the CD40 receptor has been established [1]. Atherosclerosis, a chronic inflammatory disease of the large arteries and its associated clinical manifestations, such as coronary heart disease (CHD) or cerebrovascular diseases, is the leading cause of morbidity and mortality worldwide [2]. Binding of the co-stimulatory CD40 ligand (CD154, CD40L) to its receptor CD40, especially on antigen-presenting cells, is essential for an adequate and complete immune response [3]. This interaction leads to sustained stimulation of expression of pro-inflammatory gene products in endothelial cells and their switching from a quiescent to a prothrombotic or procoagulant phenotype. This process precedes the manifestation of atherosclerosis and is intimately linked to the etiology of severe cardiovascular and rheumatic diseases [4].

A functional single nucleotide polymorphism (SNP) in the Kozak consensus sequence of the *CD40* gene (rs1883832) is closely associated with different autoimmune and inflammatory diseases by increasing protein translation efficiency and CD40 abundance on the cell surface [5]. We previously demonstrated that this SNP increases the risk of CHD in Caucasians by affecting CD40 receptor abundance on the endothelial cell surface and increasing the expression of pro-inflammatory chemokines and adhesion molecules [6]. This increased presence of CD40 on the luminal endothelial cell surface may be compensated for by a concomitant increase in expression of its soluble isoform (sCD40) or enhanced proteolytic shedding of the membrane receptor. Particularly, sCD40 levels are significantly elevated in the plasma of CC genotype patients with clinically documented CHD [6]. Thus, sCD40 may be a decoy receptor for CD40L and a potential new biomarker for the pro-inflammatory processes that emerge during the onset and accompany the progression of atherosclerosis.

In the present study, we evaluated a possible mechanism leading to an increased release of sCD40 from the endothelial cell surface of CC genotype cells under pro-inflammatory conditions caused by members of the tumor necrosis factor (TNF) superfamily. ADAM17 is a member of the ADAM (a disintegrin and metalloprotease domain) membrane-anchored proteinase family implicated in various biologic processes and associated with several chronic inflammatory diseases [7]. ADAM17 has over 80 substrates ranging from cytokines and growth factors to cell adhesion molecules and co-stimulatory receptors, including CD40 [8]. The maturation and activity of ADAM17 require the adapter proteins iRhom1 and iRhom2 encoded by the *RHBDF1* and *RHBDF2* genes, respectively [9]. To our knowledge, the regulation of the release of the sCD40 receptor from human endothelial cells under pro-inflammatory conditions has not been evaluated so far. Finally, we determined the correlation of plasma sCD40 levels in patients with CHD with other biomarkers of low-grade systemic inflammation in cardiovascular disease.

## 2. Material and Methods

Unless stated otherwise, all chemicals were purchased from Sigma-Aldrich, Taufkirchen, Germany.

### 2.1. Cell Culture and Treatments

Human umbilical vein endothelial cells (HUVEC) were isolated from freshly obtained umbilical cords provided by local hospitals with permission of the Ethics Committee (S-383/2013). Cell culture plates CellStar™ were obtained from Greiner Bio-One, Germany. Cell culture media and supplements were purchased from PromoCell, Germany. Cultivation was carried out as previously described [10]. Only cells carrying the CC genotype of the *CD40* gene (rs1883832) in passage one were used for all experiments. HUVEC were stimulated with 200 ng/mL sCD40L (Enzo Life Science, Lörrach, Germany) or 100 U/mL TNFα (Biomol, Hamburg, Germany). Messenger RNA and protein levels were examined after 8 and 24 h, respectively. Soluble CD40 protein levels were determined in the cell culture supernatant after 24 h. Expression of MCP-1 was examined after 12 h of incubation with TAPI-0 following another 8-h or 12-h stimulation for mRNA or protein detection, respectively. For ADAM17 inhibition, the medium was supplemented with 30 µmol/L TAPI-0 (Tocris, Wiesbaden, Germany) dissolved in DMSO 1 h before the stimulation started. The final concentration of DMSO in the medium was 0.1% (*v*/*v*), and the controls received DMSO only.

### 2.2. DNA Extraction and Genotyping

According to the HGVS nomenclature (https://varnomen.hgvs.org/bg-material/simple/, accessed on 11 July 2023), based on the genomic reference sequence, the analyzed −1T>C *CD40*: rs188382 polymorphism of this study is NG_007279.1:g.5077T>C. DNA was extracted from the accompanying umbilical artery by digestion and precipitation [6]. The tetra-primer amplification refractory mutation system-polymerase chain reaction (ARMS-PCR) was used with the following primers to identify the genotypes of the −1T>C SNP of the *CD40* gene: outer forward primer, GGACCGCGATTGGTCTTTGAAGACCCCG; outer reversed primer CACCCACCCTCCTGC-CCCCACAAAAATC; T-allele inner forward primer, TGGTCCTGCCGCCTGGTCTCACCTCTCT; C-allele inner reversed primer, GACGCACTGCAGAGGCAGACGAACCCTG. Depending on the genotype, agarose gel electrophoresis identified different DNA fragments [6]. 

### 2.3. Human Samples

Human blood samples were taken from 92 patients with clinically documented CHD following written consent under the ethics vote S-390/2011 at the German Heart Center, Munich. All samples were collected according to a protocol compliant with the Declaration of Helsinki. The diagnosis of CHD was confirmed by coronary angiography. Additional inclusion criteria were a lack of confounding chronic inflammatory conditions, including rheumatoid arthritis and inflammatory bowel disease. The clinical data provided for these patients included cardiovascular risk factors, kidney function, and levels of hs-troponin T and hs-CRP. Characteristics of the population are displayed in Table 1.

### 2.4. Quantitative PCR

Quantitative PCR analysis of relative mRNA expression was conducted as described previously [10]. The RNeasy Kit (Qiagen, Hilden, Germany) was used for RNA purification with effective elimination of genomic DNA. The Thermo Scientific™ NanoDrop™ One Microvolume UV–Vis Spectrophotometer was used to determine the purity of RNA samples with an A260/A280 purity ratio of 2.0 for pure RNA before reverse transcription and a quantitative PCR assay was set up. The following primer pair was used for ADAM17 (GenBank Acc: NM_003183; forward 5′-tta ttg gtg gta gca gat -3′, reversed 5′-tgt tcc gat aga tgt cat -3′). The primer sets for VCAM1, E-selectin, MCP-1, and CD40 corresponded to those previously published [6]. GAPDH was used as a housekeeping gene [6]. The GAPDH primer (for 5′-CCACTCCTCCACCTTTGAC-3′, Rev 5′-ACCCTGTTGCTGTAGCCA-3′) specifically recognizes the GAPDH transcript variants 1 (NM_002046.7), 2 (NM_001256799.3), 3 (NM_001289745.3), 4 (NM_001289746.2), and 7 (NM_001357943.2). The ΔCP relative quantification method described by Pfaffl (2001) was employed [11].

### 2.5. Western Blot, ELISA, and FACS

Protein detection by Western blot, ELISA, and FACS was done according to standard protocols. Western blot analysis was performed as described previously [10]. Briefly, protein extracts (30 µg total per lane) were separated by denaturing 10% SDS-polyacrylamide gel electrophoresis and then transferred to a polyvinylidene difluoride transfer membrane (Millipore, Burlington, MA, USA). The following primary antibodies were used for protein detection: CD40 (ab13545; Abcam, Cambridge, UK), iRhom1 (#144-65139; RayBiotech, Atlanta, GA, USA) and iRhom2 (MAB10048; R&D systems/biotechne, Wiesbaden, Germany). After incubation with HRP-substrate solution (Luminata™ Classico, Merck Millipore, Darmstadt, Germany), the transfer membrane was placed in a chemiluminescence detector (ImageQuant Las 4000 mini, GE Healthcare GmbH; Solingen, Germany). Immunoreactive band signals were quantified via densitometry using the ImageQuant software (TL 8.1, GE Healthcare GmbH, Solingen, Germany) and normalized to the internal loading control protein, β-actin.

Soluble CD40, soluble VCAM-1 (sVCAM), and MCP-1 antigen concentrations in supernatants of cultured HUVEC were measured after 24 h of culture using a commercially available ELISA assay (Quantikine ELISA, R&D Systems/biotechne, Wiesbaden, Germany). In the human plasma samples, IL-6, sCD40 (both R&D systems/biotechne, Wiesbaden, Germany), and hs-CRP (AVIVA Systems Biology, San Diego, CA, USA) ELISA were used according to the manufacturer’s instructions.

For flow cytometry detection of CD40 and ADAM17 cell surface abundance, HUVEC (1 × 10^6^ cells) were detached with Accutase solution, washed with FACS buffer [6] followed by incubation with the appropriate antibodies (Alexa Fluor^®^ 488-mouse anti-human TACE/ADAM17 ectodomain, R&D Systems/biotechne, Wiesbaden, Germany, Cat#FAB9301G; Alexa Fluor^®^ 647- mouse anti-human CD40, Biolegend/BIOZOL, Hamburg, Germany, Cat#334311) and their respective IgG controls. Unstained and untreated cells were used as controls. At least 10,000 cells were analyzed in every experiment with a BD FACSCalibur flow cytometer (Becton Dickinson, Heidelberg, Germany) using the BD FACSDiva version 6 software. Results are displayed as mean fluorescence intensity (MFI) values and normalized to the DMSO control.

### 2.6. Statistical Analysis

The GraphPad Prism 9.4.1 software (GraphPad Software, Boston, MA, USA) was used for statistical tests. Values are expressed as mean ± standard deviation (mean ± STD) and are mostly normalized to a control group. For data encompassing three groups or more, the Shapiro–Wilk test was used to test for normality. The unpaired Student’s *t*-test was used for two-group comparisons, and the one-sample *t*-test for values related to the normalized control sample. Pearson correlation coefficient r was determined. If not stated otherwise, the given *n*-number indicates individual biological replicates. *p*-values < 0.05 were considered statistically significant and are denoted on the graphs.

## 3. Results

### 3.1. ADAM17-Mediated Ectodomain Shedding of CD40 and VCAM

**Figure 1:**
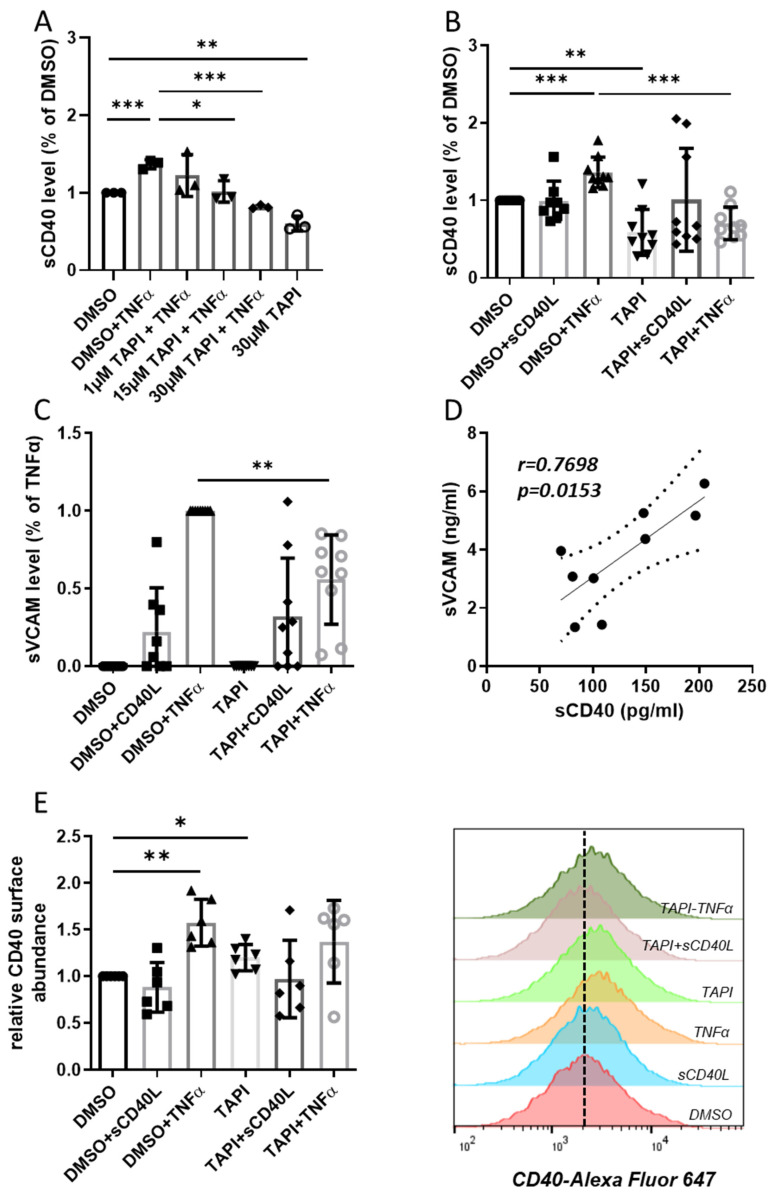
Effect of inhibition of ADAM17 on the release of sCD40 and sVCAM from HUVEC. Stimulations were carried out with sCD40L (200 ng/mL) or TNFα (100 U/mL), ADAM17 was optionally inhibited by 30 µmol/L TAPI-0. (**A**) The dose–response curve with 1–30 µmol/L TAPI-0 shows the effect of ADAM17 inhibition on sCD40 release in the conditioned medium. Bars represent the mean ± STD (*n* = 3). The TNFα-induced release of (**B**) sCD40 and (**C**) sVCAM into the supernatant of HUVEC was reduced after applying TAPI-0. Bars represent the mean ± STD (*n* = 9). (**D**) Levels of sCD40 and sVCAM correlated strongly in the supernatant of TNFα-stimulated HUVEC (r = 0.77; *p* = 0.01). (**E**) Relative CD40 protein surface abundance upon HUVECs treatment determined by flow cytometry analyses of CD40 displayed as mean fluorescence intensity normalized to DMSO control. Bars represent the mean ± STD (*n* = 6). The *p*-values are indicated on the graphs. * *p* < 0.05, ** *p* < 0.01, *** *p* < 0.001.

An initial concentration–response curve revealed that 30 µmol/L of the ADAM17 and metalloproteinase inhibitor TAPI-0 is sufficient to reduce TNFα-induced sCD40 release (136%) below control levels (Figure 1A). Basal sCD40 release into the conditioned medium without stimulation was reduced by TAPI-0 even further to 58% of the DMSO control (Figure 1A).

In another set of experiments, TAPI-0-mediated ADAM17 inhibition resulted in an almost identical decrease in basal sCD40 release into the supernatant (Figure 1B). Stimulation with TNFα raised sCD40 release to the same extent as in Figure 1A and was even more effectively downregulated following ADAM17 inhibition (Figure 1B). Neither stimulation with sCD40L alone nor in combination with TAPI-0 had any effect on sCD40 release into the conditioned medium compared to the DMSO control. In contrast, TAPI-0 diminished sVCAM release from HUVEC stimulated with TNFα (Figure 1C). Soluble CD40L-induced sVCAM release was not affected following the inhibition of ADAM17. Moreover, we noted a strong correlation between sCD40 and sVCAM levels in the conditioned medium of the TNFα-stimulated HUVEC (Figure 1D).

Accordingly, we assumed an increased abundance of CD40 on the surface of the endothelial cells upon inhibition of ADAM17 due to reduced shedding. In fact, inhibition of ADAM17 by TAPI-0 without stimulation significantly increased CD40 protein levels by 20% (Figure 1E). Exposure to sCD40L did not affect the abundance of CD40 on the endothelial cell surface, while this was elevated in the presence of TNFα by 58% compared to control. Concomitant treatment with TAPI-0 had no discernible impact on TNFα-dependent CD40 surface abundance (Figure 1E). Thus, upon stimulation with TNFα, enhanced ADAM17 activity apparently causes an increased shedding of sCD40, resulting in a maintenance of CD40 receptor density on the endothelial cell surface.

### 3.2. Differential Inflammatory Activation of the ADAM17 Regulators iRhom1 and 2

**Figure 2:**
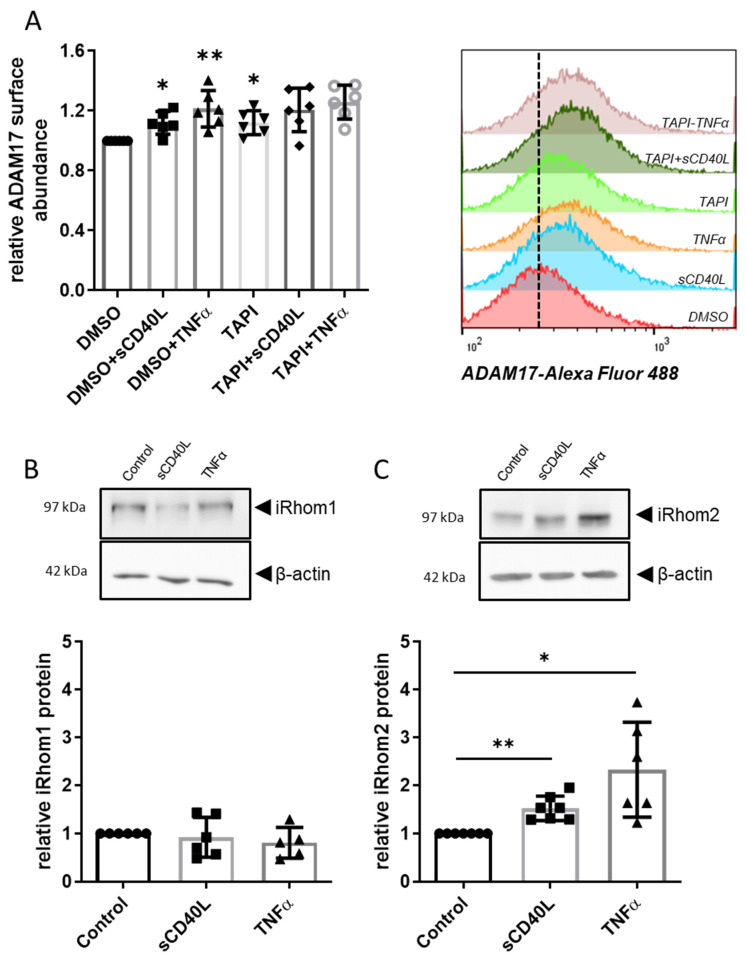
Differential induction of the ADAM17 regulators iRhom1 and 2 in HUVEC. (**A**) Flow cytometry analyses of ADAM17 surface expression displayed as mean fluorescence intensity normalized to DMSO control. Stimulations were carried out with TNFα (100 U/mL) and sCD40L (200 ng/mL), ADAM17 was optionally inhibited by 30 µmol/L TAPI-0. Bars represent the mean ± STD (*n* = 6). (**B**) iRhom1 and (**C**) iRhom2 protein expression in HUVEC after stimulation with sCD40L (200 ng/mL) or TNFα (100 U/mL). Exemplary Western blot analyses and statistical summary. Bars represent the mean ± STD (*n* = 6). *p*-values are indicated on the graphs. * *p* < 0.05, ** *p* < 0.01.

Stimulation with sCD40L or TNFα moderately but significantly enhanced the abundance of ADAM17 on the endothelial cell surface by 10 and 21%, respectively (Figure 2A). Exposure to TAPI-0 alone resulted in a marginal rise of ADAM17 surface abundance by 12%. TAPI-0 did not affect the changes in ADAM17 abundance elicited by CD40L or TNFα (Figure 2A). Following sCD40L or TNFα stimulation, iRhom2 protein abundance increased 1.5-fold and 2.4-fold, respectively (Figure 2C), while iRhom1 protein levels remained unchanged (Figure 2B).

### 3.3. Impact of TAPI-0-Mediated ADAM17 Inhibition on CD40 Signaling

The results so far demonstrated that ADAM17 inhibition by TAPI-0 leads to a decrease in baseline shedding of sCD40 and a corresponding rise in the abundance of CD40 on the surface of the endothelial cells. Pro-inflammatory conditions, i.e., stimulation with TNFα, increased CD40 on the endothelial cell surface and enhanced shedding of sCD40 simultaneously. This enhanced release of sCD40 was reduced by inhibiting ADAM17 activity.

**Figure 3:**
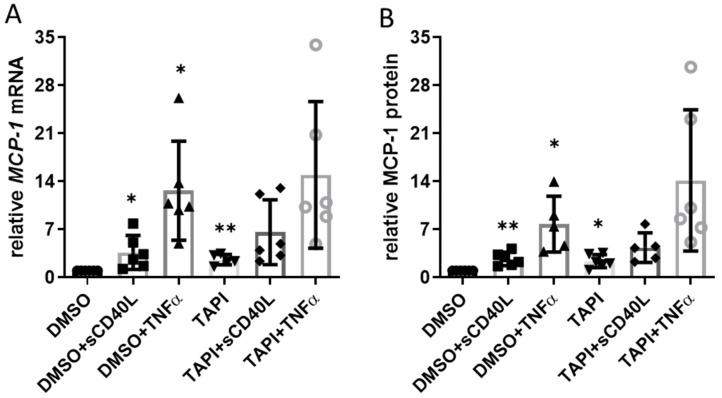
Impact of ADAM17 inhibition on CD40 signaling. HUVEC were incubated with 30 µmol TAPI-0 or DMSO for 12 h and then stimulated with sCD40L (200 ng/mL) or TNFα (100 U/mL). (**A**) MCP-1 mRNA expression (after 8 h) and (**B**) MCP-1 protein in the supernatant (after 24 h) were determined by RT-PCR and ELISA, respectively. Data were normalized to DMSO control, and *p*-values are indicated on the graphs. * *p* < 0.05, ** *p* < 0.01.

Interaction of endothelial cell CD40 with sCD40L elicits a profound increase in the expression of the pro-inflammatory chemokine monocyte chemoattractant protein-1 (MCP-1/CCL2) [10]. Therefore, we studied whether ADAM17 inhibition also impacts the expression of the *CCL2* gene and the release of MCP-1 from the CC genotype HUVEC as a read-out for CD40 functionality. Without sCD40L or TNFα, TAPI-0 increased MCP-1 expression on the mRNA (2.6-fold, Figure 3A) and protein level (2.4-fold, Figure 3B) to a similar extent. Stimulating the cells with sCD40L or TNFα resulted in a substantial further increase in MCP-1 mRNA and protein levels. 

However, the abundance of CD40 on the cell surface remained constant in the presence of sCD40L or TNFα when the HUVEC had been pre-treated with TAPI-0 (Figure 1E). This finding was not surprising because concomitant treatment with TAPI-0 had no additional effect on MCP-1 mRNA or protein in response to stimulation with TNFα or sCD40L (Figure 3).

### 3.4. Correlation of sCD40 Plasma Levels with Other Biomarkers for CHD

**Figure 4:**
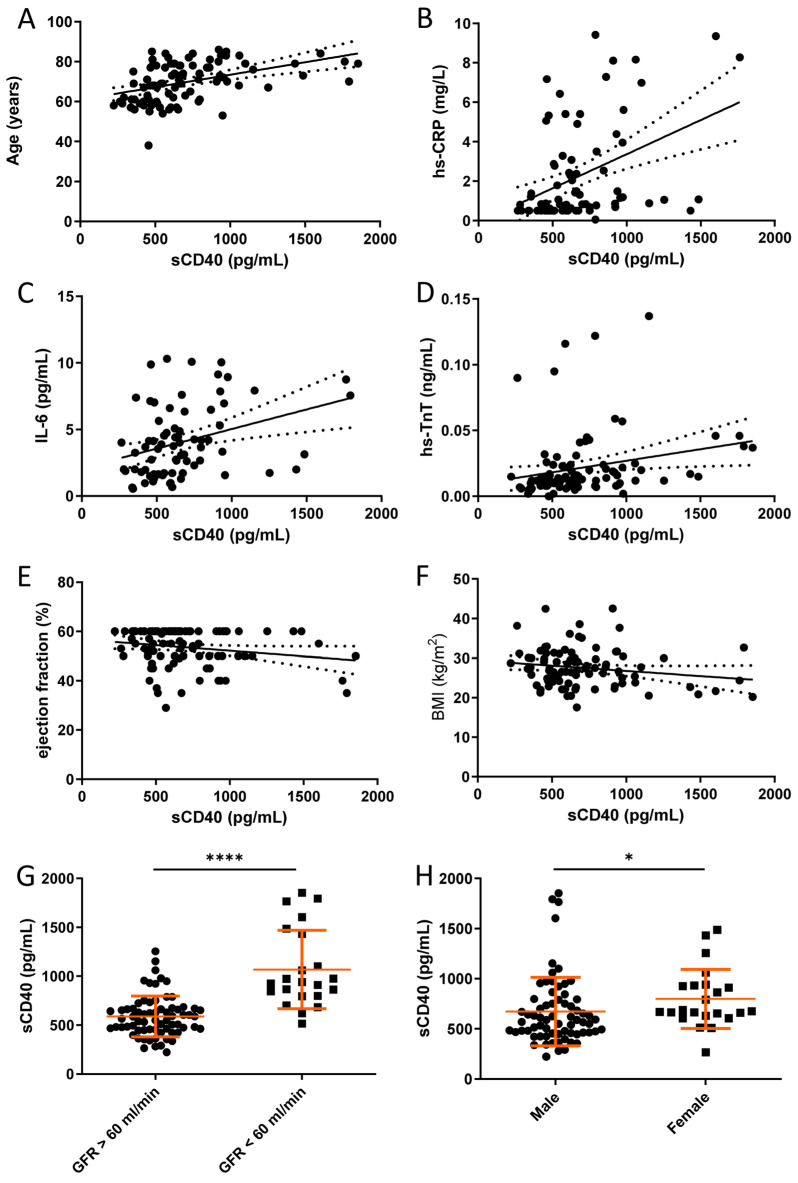
Correlations for sCD40 as a biomarker for CHD in plasma samples. (**A**–**F**) Data were analyzed by Pearson’s correlation. The sCD40 level showed an age dependency (**A**) (r = 0.43; *p* < 0.0001). We observed a medium positive correlation of sCD40 to hs-CRP (**B**) (r = 0.41; *p* = 0.0001) and IL-6 (**C**) (r = 0.34; *p* = 0.0032), as well as a small correlation to hs-TnT (**D**) (r = 0.23; *p* = 0.02). The small negative correlation to the ejection fraction (**E**) did not quite reach the level of significance (r = −0.2; *p* = 0.059). No correlation was observed with the body mass index of female and male patients (**F**). (**G**,**H**) Effects of glomerular filtration rate (GFR) and gender on plasma sCD40 levels in patients with CHD. *p*-values are indicated on the graphs. * *p* < 0.05, **** *p* < 0.0001.

Finally, we determined possible correlations of sCD40 plasma levels with other biomarkers of inflammation and clinical parameters in patients with CHD (Figure 4). We noted a medium positive correlation between sCD40 plasma levels and the age of the patients (Figure 4A) as well as with the inflammation biomarkers hs-CRP and IL-6, respectively (Figure 4B,C). A weak positive correlation was found for sCD40 plasma levels and the cardiac biomarker high-sensitivity troponin T (Figure 4D). At the same time, there was a trend (*p* = 0.0593) towards a negative correlation with left ventricular ejection fraction (Figure 4E). There was no correlation of sCD40 plasma levels with the body mass index of female and male patients (Figure 4F). Finally, a lower glomerular filtration rate was associated with increased sCD40 levels in the plasma of patients with CHD (Figure 4G), and female patients had higher sCD40 plasma levels than their male counterparts (Figure 4H). 

## 4. Discussion

Here, we present a mechanism by which the CD40 receptor is shed from the endothelial cell surface by the protease ADAM17 giving rise to the soluble CD40 isoform and limiting CD40 downstream signaling. Moreover, sCD40 plasma levels may represent an additional robust biomarker for patients with CHD. 

Whereas the role of ADAM17 in the processing of CD40 has been studied in B cells [12], its role in endothelial cells remains so far unexplored. To this end, we focused on endothelial cells harboring the pro-inflammatory CC genotype of the −1T>C SNP of the *CD40* gene [6]. We showed that TNFα induces the release of sCD40 from the surface of the endothelial cells and that this effect is prevented by inhibiting ADAM17 activity. In contrast, stimulation with sCD40L did not elicit any sCD40 release suggesting that another mechanism such as internalization of CD40 [13] may compete with its shedding from the endothelial cell surface, thereby impeding the formation of sCD40.

Moreover, our data suggest that ADAM17 is primarily responsible for forming sCD40 under pro-inflammatory conditions via shedding the membrane-bound CD40 receptor. We found a similar pattern for the adhesion molecule VCAM-1 and its shedding by ADAM17 from the endothelial cell surface. Hence, the abovementioned notion aligns with and expands previous studies [14]. The strong correlation between the levels of sCD40 and sVCAM-1 in the supernatant of the cultured HUVEC stimulated with TNFα emphasizes the significance of ADAM17 in shedding CD40 from the endothelial cell surface. Nevertheless, it needs to be considered that splice variants of CD40 expressed by human endothelial cells may also be partially responsible for an increase in the formation of sCD40 [15,16]. 

Flow cytometry analyses confirmed an increased abundance of CD40 on the surface of the cultured HUVEC following inhibition of ADAM17 in the absence of any stimulus. Under pro-inflammatory conditions, CD40 receptor density on the endothelial cell surface remained rather constant and did not seem to be influenced by blocking ADAM17 activity. This observation raised the question of whether there is an impact of ADAM17 on CD40 downstream signaling. Using the expression of the *CCL2* gene (encoding the chemokine MCP-1) as a read-out, there was no significant upregulation of the CD40 signal transduction pathway secondary to inhibition of ADAM17. However, we also observed an increase in *CCL2* expression after exposure to TAPI-0. One possible explanation is endogenous activation of TNF receptor 2 (TNFR2) signaling through membrane-bound TNFα because ADAM17 can cleave membrane-bound TNFα and TNFR2, thus preventing undue activation of TNFR2 in vascular cells [17].

Furthermore, we found that the abundance of ADAM17 on the endothelial cell surface increases upon exposure to sCD40L or TNFα, indicating a possible negative feedback mechanism. This is in line with studies describing an anti-inflammatory role of ADAM17 by shedding adhesion molecules and TNF receptors from the endothelial cell surface, thereby impeding leukocyte adhesion and pro-inflammatory downstream signaling [14,17,18]. A similar mechanism was shown for TNFα and interferon-γ (IFNγ)-induced shedding of the junctional adhesion molecule JAM-A by ADAM17 [19] with the released sJAM-A acting as a decoy for neutrophils limiting their transmigration. Soluble CD40 is also known to act as a decoy neutralizing CD40L [16]. Therefore, it would be interesting to verify this possible role of sCD40 as a decoy for CD40L-expressing cells such as, e.g., platelets [20]. 

We further evaluated the role of the ADAM17 regulators iRhom1 and iRhom2. While expression of iRhom2 was upregulated following stimulation with sCD40L or TNFα, expression of iRhom1 remained unaffected. This indicates constitutive expression of *RHBDF1*, while *RHBDF2* expression seems influenced by pro-inflammatory endothelial cell activation. These findings are in line with previous reports indicating that TNFα and IFNγ upregulate *RHBDF2* expression. In contrast, *RHBDF1* expression is rather controlled by unidirectional shear stress giving rise to increased formation of endothelial cell nitric oxide [21]. Therefore, it would be interesting to elucidate the interrelation between unidirectional vs. oscillatory shear stress, *RHBDF1* expression, and the shedding of CD40 from the endothelial cell surface.

Following our previous work [22], we investigated whether sCD40 is a reliable biomarker for CHD. While sCD40L as a biomarker has already been extensively studied [23,24], little is known about the significance of sCD40 in this context. The relevance of the inflammatory status for cardiovascular diseases has been well documented [25]. High-sensitivity CRP and IL-6 as markers of vascular inflammation allow risk stratification and prediction of cardiovascular events [26]. The correlation of sCD40 with these markers suggests a similar role for sCD40. The sequelae of atherosclerosis, such as cardiovascular events and impairment of cardiac function [27], also seem to be associated with sCD40 plasma levels, as emphasized by the correlation between sCD40 and high-sensitive troponin T, and the trend towards an inverse correlation between sCD40 and left-ventricular ejection fraction.

Interestingly, sCD40 plasma levels increase with age, indicating an enhanced ADAM17-dependent shedding of CD40 from the luminal endothelial cell surface. This observation is consistent with studies demonstrating an age-dependent increase of ADAM17 activity in endothelial cells in the adipose tissue of obese individuals [28]. In line with our data, an association of sCD40 plasma levels with the risk of stroke, myocardial infarction and atherosclerotic plaque burden in the carotid arteries has recently been established [29]. Soluble CD40 was found to be the strongest predictor of atherosclerotic plaque burden in the carotid artery bifurcation, among several other markers tested [30]. A role for sCD40 as a biomarker was also suggested in several other inflammatory diseases such as, e.g., ulcerative colitis [31], systemic sclerosis [32], and lupus erythematosus [33].

Some limitations for endothelial cell-derived sCD40 as a biomarker must be considered. Even though we can almost exclude contamination with genomic DNA due to the RNA isolation including purity control method used, GADPH may not be the ideal housekeeping gene for PCR. Genomic DNA contamination of RNA samples containing many GAPDH pseudogenes with strong global homology to the mRNA can lead to inaccurate measurement of gene expression by reverse transcription quantitative real-time PCR.

Our data indicate that glomerular function strongly affects sCD40 plasma levels. Substantial impairment of renal function has been shown to raise sCD40 levels in the plasma of patients undergoing hemodialysis [34]. Ultimately, the origin of the elevated sCD40 levels in the plasma of patients with CHD remains unclear, too. Although endothelial cells seem to play an important role therein, a substantial release of sCD40 from the surface of antigen-presenting cells such as B cells, particularly monocyte/macrophages, may also occur in atherosclerosis. Furthermore, our study only addressed sCD40 plasma levels at one and in view of the stage of atherosclerosis/CHD somewhat variable time point. A prospective study linking sCD40 plasma levels with atherosclerotic plaque burden and cardiovascular events would be desirable. 

Nonetheless, there are also certain opportunities for using sCD40 as a biomarker for CHD. As part of the Atherosclerosis Risk In Communities (ARIC) study, a possible use of the adhesion molecules ICAM-1 and E-selectin as biomarkers for CHD and atherosclerosis in the carotid arteries was demonstrated [35]. In another study, VCAM-1 was identified as a marker for peripheral artery disease [36]. Similar to CD40, both endothelial cell adhesion molecules are processed and cleaved by ADAM17 [8]. Therefore, investigating a panel that records a combination of sCD40 and other proteins cleaved by ADAM17 may help assess cardiovascular risk more adequately. A corresponding score, which monitors ADAM17 activity by measuring the processed proteins, was developed to predict cardiovascular events [37]. Our data show a strong correlation between sCD40 and sVCAM-1 concentrations in the supernatant of human endothelial cells stimulated with TNFα underpinning this thought on the cell culture level. The application of sCD40 as a novel biomarker for CHD that stands out from other markers of inflammation may come from its combination with genotyping for the -1T>C SNP of the *CD40* gene, which we recently established as a genetic risk factor for CHD. This combination may thus allow for a more robust prediction of cardiovascular events in the future.

## Figures and Tables

**Table 1:** Clinical characteristics of the study population. Values are represented as means ± standard deviation.

Groups	CHD (*n* = 92)
Age (years)	69.8 ± 9.7
Sex (M/F)	69/23
Smoker (%)	48.9
Hypertension (%)	93.5
Diabetes (%)	47.9
Hypercholesterolemia (%)	97.8
Body mass index (kg/m^2^)	97.8 ± 4.9

## Data Availability

All data generated or analyzed during this study are included in this article. Further inquiries can be directed to the corresponding author.
